# Cognitive decline after ventriculoperitoneal shunt surgery for normal pressure hydrocephalus

**DOI:** 10.1111/psyg.13117

**Published:** 2024-03-31

**Authors:** Ying‐Chih Liao, Chia‐Liang Wu

**Affiliations:** ^1^ Department of Psychiatry Taipei Veterans General Hospital Taiwan

Dear Editor,

Normal pressure hydrocephalus (NPH) is caused by excess cerebrospinal fluid (CSF) in the brain ventricles. The classic triad of NPH includes gait disturbance, urinary incontinence, and cognitive dysfunction. Inserting a ventriculoperitoneal shunt (VPS), to drain excess CSF from the brain to the abdomen, is the first‐line treatment for individuals with NPH. Currently, the risk–benefit assessment indicates that this invasive procedure improves outcomes compared to conservative treatment.^1^ Up to two‐thirds of patients with NPH show positive outcomes after VPS placement when appropriately selected.^2^, ^3^ However, the outcomes of cognitive function after treatment remain controversial. Here, we report a case of cognitive decline following VPS surgery for NPH.

A 78‐year‐old Asian woman presented with gait disturbance accompanied by urinary incontinence that had persisted for several months. She had a cerebrovascular accident (CVA) approximately 19 years previously and remained fair in activities of daily living. Impaired cognition was noted when she visited the doctor, and a series of examinations were performed. The Mini‐Mental Status Examination (MMSE) score was 12 (<cut‐off point: 23/24), and the Clinical Dementia Rating (CDR) was 2, indicating moderate cognitive impairment. A computed tomography (CT) scan revealed an ageing brain with ventriculomegaly. The symptoms improved after lumbar puncture. Based on these findings, NPH was diagnosed. The neurosurgeon then decided to perform VPS placement, and the surgery proceeded smoothly. After surgery, gait symptoms and urinary incontinence markedly improved; however, a significant decline in cognitive function was observed within 3 months, and a brain CT scan was performed again. This report revealed the presence of a 6 mm high‐density lesion in the pons. Her vital signs were relatively stable, and the size of the lesion decreased, according to a subsequent brain CT scan. Therefore, pontine haemorrhage was less likely and calcification was highly suspected. Subsequently, she regularly visited our psychiatric outpatient department due to declining cognition. Psychological testing showed that the MMSE score was 2 and the CDR score was 3. There were no significant behavioural or psychological symptoms of dementia under the current psychotropic regimen of risperidone 0.5 mg twice daily and memantine 10 mg daily. She was wheelchair‐dependent, mostly due to weakness in the bilateral lower extremities.

This case report describes cognitive decline after VPS surgery for NPH. Currently, VPS remains the standard and optimal treatment strategy for individuals with NPH based on prominent treatment effects with an acceptable complication rate. Previous studies have reported that negative prognostic factors include moderate or severe cognitive impairment and the presence of dementia for more than 2 years.^4^ Therefore, the selection of appropriate patients could improve the overall clinical outcomes and diminish unfavourable outcomes. In this patient, cognitive impairment may have resulted from a CVA 19 years prior, and she had moderate cognitive impairment before surgery. These factors may be associated with a negative surgical response. In conclusion, a comprehensive approach to risk–benefit assessment before surgery is important.

## FUNDING INFORMATION

There is no funding source.

## DISCLOSURE

The authors declared no conflicts of interest associated with this manuscript.

## ETHICS STATEMENT

The patient described in the study provided informed consent.
